# A MicroRNA Component of the Neoplastic Microenvironment: Microregulators with Far-Reaching Impact

**DOI:** 10.1155/2013/762183

**Published:** 2012-12-04

**Authors:** Xiaolei Li, Zhiqiang Wu, Xiaobing Fu, Weidong Han

**Affiliations:** Department of Molecular Biology, Institute of Basic Medicine, School of Life Sciences, Chinese PLA General Hospital, Beijing 100853, China

## Abstract

The interplay between tumor cells and their microenvironment plays a pivotal role in tumor development and progression. Although a growing body of evidence has established the importance of the tumor microenvironment, an understanding of the crosstalk between its components and cancer cells remains elusive. The pathways triggered by microenvironmental factors could modulate cancer-related gene transcription, also affecting small noncoding RNAs, microRNAs, which have emerged as key posttranscriptional regulators of gene expression, directly involved in human cancers. Although microRNAs regulate most biological mechanisms, their role in the tumor microenvironment has only recently become the focus of intense research. In this paper, we focus on the intertwined connection between the tumor microenvironment and aberrant expression of microRNAs involved in carcinogenesis. We also discuss the emerging roles of microRNAs in the tumor microenvironment as it relates to cancer progression. We conclude that microRNAs are critical for our understanding of the development of cancer, and that targeting microRNA signaling pathways in the microenvironment as well as in tumor cells opens new therapeutic avenues to the global control of cancer.

## 1. Introduction

**Figure 1:**
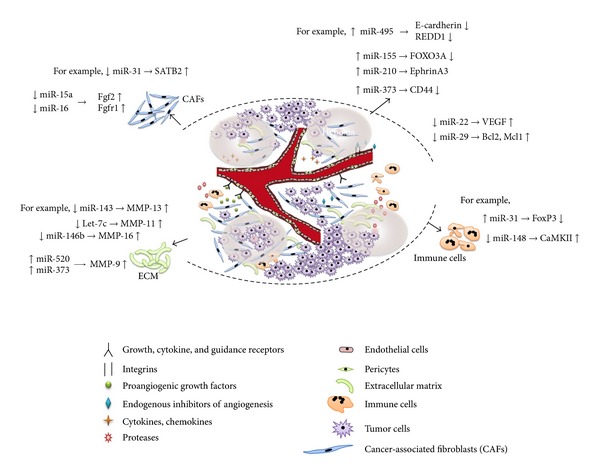
Characteristics of the tumor microenvironment or niche, and validated microRNAs and their target genes in the microenvironment. The tumor microenvironment is a complex scaffold of extracellular matrix and various cell types. In addition to tumor cells, an assemblage of distinct cell types, including endothelial cells, fibroblasts, and immune cells, as well as extracellular matrix molecules, contribute to tumor growth and progression. Tumor tissue is characterized by self-sufficiency in growth signals, which provides replicative immortality, and enables angiogenesis, cell invasion, and metastasis. The different cell types in the tumor can produce growth and antigrowth signals and respond to stimuli secreted by other cells. This creates a favorable microenvironment for tumor growth and spread. The influence of microRNAs on the tumor microenvironment is related to cancer progression. MicroRNAs are a critical component of the tumor microenvironment involved in invasion and metastasis of cancer cells. The deregulated expression of microRNAs in the tumor microenvironment could contribute to cancer proliferation, invasion, and metastasis. See text for more details.

The hallmarks of cancer comprise ten biological capabilities acquired during the multistep development of human tumors, which together constitute an organizing principle for rationalizing the diversities and complexities of neoplastic disease [1, 2]. They include sustaining proliferative signaling, evading growth suppressors, resisting cell death, enabling replicative immortality, inducing angiogenesis, activating invasion and metastasis, genome instability and mutation, tumor promoting inflammation, reprogramming energy metabolism, and evading immune destruction (reviewed in [1]). Over the past decade, solid tumors have increasingly been recognized as “organs” whose complexity approaches and may even exceed that of normal healthy tissues [3]. When viewed from this perspective, the biology of a tumor can only be understood by studying the individual specialized cell types within it that develop during the course of multistep tumorigenesis. Solid tumors are more than just insular masses of proliferating cancer cells. They are complex tissues composed of multiple distinct cell types that participate in heterotypic interactions with one another. This depiction contrasts starkly with the earlier, reductionist view of a tumor as nothing more than a collection of relatively homogeneous cancer cells, whose entire biology could be understood by elucidating the cell-autonomous properties of these cells (reviewed in [1]). Until recently, much of the cellular heterogeneity within tumors was found in their stromal compartments. Notably, solid tumors contain a repertoire of recruited, ostensibly normal cells, which form tumor-associated stroma, and are active participants in tumorigenesis rather than passive bystanders. As such, these stromal cells contribute to the acquisition of hallmark traits by creating the “neoplastic microenvironment,” which exhibits another dimension of complexity. These nonmalignant cells include several different types such as tumor-associated fibroblasts, myofibroblasts, adipocytes, resident epithelial cells, endothelial cells, pericytes, and immune inflammatory cells (Figure 1).

As the carcinoma progresses, the microenvironment of cancer cells coevolves into an activated status through continuous paracrine communication and the concurrent activity of stromal and cancer cells, thus creating a dynamic signaling circuitry in which diverse molecules cooperate to promote tumor expansion and invasiveness, ultimately leading to the development of a fatal disease [4]. The dynamic crosstalk and interactions between neoplastic cells and normal cells in the microenvironment regulate the production of critical factors that determine whether cancer progression will occur by directly promoting cellular transformation and metastasis. Tellingly, although the cells within the tumor microenvironment do not undergo malignant transformation, dramatic changes in their gene expression profiles distinguish them from their normal counterparts [5, 6]. As pleiotropic modulators of gene expression, microRNAs (miRNAs) participate in a wide variety of physiological and pathological cellular processes as well as playing a role in the development of many diseases, including cancer [7]. Over the last several years, important roles for miRNAs during all stages of cancer progression have been established, including their involvement as tumor oncogenes or suppressors and the regulation of metastatic spread [8]. However, the role of miRNAs in the tumor microenvironment and their association with the development and progression of tumors have only recently become a focus of intense research. In the present study, we examine how miRNAs are misregulated in the tumor microenvironment and how this affects tumor biology. Understanding the miRNA-regulated changes occurring in the tumor microenvironment may provide new avenues for intervention and contribute to the global control of many cancers.

## 2. Emerging Roles of MicroRNAs in Response to Hypoxia

Hypoxia is an essential feature of the neoplastic microenvironment and contributes to increased invasion, poor prognosis, and resistance to conventional therapy [9]. During the past decade, gene induction by low oxygen was the focal point of hypoxia research. However, more recently, the study of gene expression regulated by a hypoxic microenvironment has become a focus of intense research. Furthermore, the influence of miRNAs on homeostatic regulation and in particular hypoxic responses has generated increasing interest [10, 11] (Figure 1). Recent studies have identified a signature of hypoxia inducible miRNAs that includes miR-21, 23a, 23b, 24, 26a, 26b, 27a, 30b, 93, 103, 106a, 107, 125b, 181a, 181b, 181c, 192, 195, 210, and 213 [10, 12]. Data from recent studies have indicated that miR-210, 30b, 93, and 181b were consistently induced in response to low oxygen with some notable similarities [13–15]. The different cellular backgrounds included and the microarrays used resulted in a significant number of miRNAs that differed between the studies. For instance, some of the miRNAs, including miR-429, 498, 563, 572, 628, and 637, have been identified as hypoxia-induced using a different type of microarray [14]. In addition to the miRNAs that are upregulated in response to low oxygen, some miRNAs were identified as downregulated under hypoxic conditions, including miR-19a, 29b, 30e-5p, 101, 122a, 141, 186, 195, 197, 320, 374, 422b, 424, and 565 in squamous cell carcinoma cells, miR-15b, 16, 20a, 20b, 30b, and 224 in carcinoma of nasopharyngeal epithelial cells, and miR-424 in trophoblasts [13–15]. Intriguingly, there is also evidence that hypoxia-responsive miRNAs may be regulated in a more cell-specific manner. For example, let-7e, 7 g, and 7i were identified as hypoxia-inducible factors in squamous cell carcinoma [14], whereas let-7a, 7c, 7d, 7e, 7f, and 7g were downregulated by hypoxia in nasopharyngeal carcinomas [13].

A significant proportion of the hypoxia-regulated miRNAs are also overexpressed in human solid tumors, suggesting a role in tumorigenesis and/or tumor progression [10]. The upregulation of miR-495 in breast cancer cells was shown to suppress E-cadherin expression to promote cell invasion, and inhibit REDD1 expression to enhance cell proliferation under hypoxic conditions [16]. Therefore, miR-495 contributes to breast cancer cell proliferation and hypoxia resistance. miR-21 is overexpressed in glioblastoma in the presence of a hypoxic microenvironment, and modulates the expression of target genes necessary for tumor survival [17, 18]. The role of miRNAs in lung cancer was reported in a similar study. MiR-155 was elevated in lung cancers characterized by an extensive hypoxic microenvironment, and an increased level of miR-155 was found to radioprotect lung cancer cells, whereas miR-155 inhibition radiosensitizes these cells [19].

Numerous profiling and characterization studies of miRNAs have identified critical roles for noncoding RNAs in cancer. MiRNAs are involved in tumorigenicity, acting as oncogenes or tumor suppressors depending on their target genes, and their roles in solid tumors under hypoxic conditions are becoming apparent. Mechanistically, select miRNAs including miR-210, 155, 107, 21, 20a, 20b, 26, 27, 31, 373, and 495 may play a role in cancer under hypoxia conditions by acting as oncogenes [20]. Of these, miR-210 and miR-373 have been studied extensively as hypoxia-regulated miRNAs with a role in cancer [21]. Compared to the corresponding normal tissue, miR-210 is significantly upregulated in most solid tumors [22–25]. More interestingly, under normoxic conditions, the expression levels of miR-210 are reduced in tumor-derived cell lines. One reasonable interpretation of this phenomenon suggests that the tumor hypoxic microenvironment may contribute to the high levels of miR-210 found *in vivo*. Clinically, enhanced expression of miR-210 is strongly associated with poor outcome and metastatic potential [25, 26]. Similar to miR-210, miR-373 promotes tumor invasion and metastasis, as demonstrated by its overexpression in the nonmigratory and nonmetastatic phenotype MCF7 cells, which enhanced cancer cell migration and invasion *in vitro* and *in vivo *[27]. Furthermore, the expression of miR-373 has been correlated with increased cell proliferation and tumorigenesis [28]. In PC3 cells, miR-373 may promote the development of metastases from prostate cancer by targeting the E-cadherin promoter region and rapidly inducing its expression [29]. Thus, miR-373, similar to miR-210, is induced during hypoxia and acts as an oncogene, providing a link to hypoxia-induced tumor progression.

In addition to their role in cancer as oncogenes, a tumor-suppressing role under hypoxic conditions has been associated with several miRNAs including miR-15a, 16, 22, 29, 107, 145, and 519c. Decreased levels of miR-15a and miR-16 during hypoxia have been found to contribute to sustained expression of VEGF, and consequently to the promotion of angiogenesis [13]. On the other hand, cancer-associated genomic regions could be disrupted by chromosomal abnormalities, which include minimal regions of loss of heterozygosity. These regions may include genes-encoding miRNAs, confirming the connection between altered miRNA expression and cancer [30]. As an example, chromosome 13q14 deletions are found at high frequency in B-cell chronic lymphocytic leukemias (B-CLL) [30]. A minimal deleted region has been identified that includes the deleted in leukemia 2 gene (*DLEU2*) [31], which encodes a long noncoding RNA (1.0–1.8 kb) that is polyadenylated and spliced [31], and the miR-15a/16 cluster that is located intronic to *DLEU2* [30, 32]. A recent study from Klein et al., showed that deletion of the miR-15a/16 cluster in B cells may accelerate the G_0_/G_1_-S phase transition by causing a defect in the negative regulation of the expression of molecules critically involved in this transition [33]. Taken together, these findings suggest that the *DLEU2*/miR-15a/16 locus has a tumor-suppressor role in the B-cell lineage *in vivo*, providing a paradigm for a similar role of other sterile transcripts in human diseases. In HCT116 colorectal cancer cells, enhanced expression of miR-22 represses vascular endothelial growth factor (VEGF) production during hypoxia and inhibits endothelial cell growth and invasion [34]. Therefore, miR-22 might have an antiangiogenic effect in colon cancer. In another example, the forced expression of miR-29 sensitized hepatocellular carcinoma (HCC) cells to apoptosis under serum starvation and hypoxia conditions through a mitochondrial pathway involving Mcl-1 and Bcl-2 [35]. Hypoxia-inducible factor-1 (HIF-1) is widely considered to be one of the key regulators in cancer. Studies have suggested that miRNAs directly inhibit the expression levels of HIF-1 and participate in the regulation of tumor angiogenesis. For example, mice injected with miR-107, miR-145, and miR-519c overexpressing tumor cells showed dramatically reduced HIF-1 levels, followed by suppressed tumor angiogenesis, growth, and metastasis [36–38]. Therefore, miRNAs serve as tumor suppressors, downregulating HIF-1 expression by posttranscriptional regulation and leading to the inhibition of tumor angiogenesis, growth, and metastasis.

Hypoxia is one of the fundamental biological phenomena that are intricately associated with the progression and aggressiveness of a wide variety of solid tumors. The molecular mechanisms underlying the response to hypoxia are extremely complex, and a key role is played by HIF, which orchestrates the expression of a wide variety of genes thought to be critical for adaptation to hypoxia. The HIF transcription factors contain an oxygen-regulated *α*-subunit and a constitutively expressed *β*-subunit. Under normoxic conditions, the *α*-subunit is degraded. However, in hypoxia, HIF is stabilized and regulates the expression of a wide variety of genes, including some involved with angiogenesis and survival, all of which play pivotal roles in the development of cancer under hypoxic conditions. An important regulatory role of HIF, at least for a subset of hypoxia-inducible miRNAs under low oxygen conditions, was demonstrated using chromatin immunoprecipitation (ChIP) arrays to show that HIF binds to the promoters of regulated miRNAs [9]. However, the specific factors involved in the regulation of miRNAs that are downregulated in the hypoxic response have not yet been identified [39]. 

## 3. Regulatory Roles of MicroRNAs in Cancer-Associated Fibroblasts 

Stromal cells, including fibroblasts, endothelial cells, pericytes, and immune cells, collaborate with tumor cells to promote tumor progression [40]. Cancer-associated fibroblasts (CAFs), the most common cellular population found in the tumor microenvironment, support tumor growth and dissemination [41]. Currently, the underlying molecular mechanisms contributing to CAF-mediated tumor spreading and invasiveness are not fully understood. Genome analyses of the tumor stroma indicate that the hot spots for mutation are accumulated in the stroma much like neoplastic cells [42, 43]. However, genetic alterations are rare in CAFs, suggesting that other manners of altering gene expression profiles in these cells must exist [44]. As the “noncoding RNA revolution” continues to unfold, a large number of miRNAs are being revealed as essential for normal development as well as that of several diseases, especially cancer [7].

Several studies have indicated that certain miRNAs in CAFs are capable of regulating the process of tumor metastasis based on the secretion of growth factors (HGF, IGF, FGF, VEGF, Wnt, and TGF*β*) as well as extracellular matrix-degrading metalloproteinases (MMPs) [12] (Figure 1). A variety of miRNAs in CAFs have been suggested to act as oncogenes and shown to be upregulated in certain cancers. A previous study in colorectal cancer reported the predominant expression of miR-21 in CAFs and showed that the frequency and extent of its expression increase during colorectal cancer progression from precancerous adenoma to advanced carcinoma [45]. Clinically, high miR-21 levels in CAFs have been associated with poor prognosis in patients with breast cancer and pancreatic cancer [46–48].

In contrast to the upregulated miRNAs, certain miRNAs downregulated in CAFs were found to act as metastasis suppressors in various tumor types. A good example is miR-31, which is downregulated in CAFs derived from endometrial cancer compared to paired normal endometrial fibroblasts [49]. Functional analyses revealed that overexpression of miR-31 considerably impaired the ability of CAFs to stimulate tumor cell migration and invasion by directly targeting the homeobox gene *SATB2*, which encodes a nuclear matrix-attachment protein responsible for chromatin remodeling and transcriptional regulation (reviewed in [49]). In addition, downregulation of miR-15a and miR-16 in CAFs in the prostate cancer microenvironment promoted tumor growth and progression by suppressing the posttranscriptional repression of Fgf-2 and its receptor Fgfr1, which enhance tumor-cell survival, proliferation, and migration by acting on both stromal and tumor cells [50]. As mentioned above, these findings revealed that miRNAs act as suppressors of cell-autonomous metastatic phenotypes. Another interesting miRNA, miR-126, is silenced in a variety of common human cancers and suppresses metastatic endothelial-cell recruitment in non-cell-autonomous cancer progression processes through the coordinated targeting of *IGFBP2*, *PITPNC1,* and* MERTK*, which are novel proangiogenic genes and biomarkers of human metastasis [51].

The crosstalk between CAFs and immune cells in the tumor microenvironment has been well established. The exchange of miRNAs between these cellular components may be an important means of cell-cell communication. For instance, miR-31 inhibits expression of the forkhead box P3 (FoxP3) transcription factor that is necessary for active Treg function, providing an additional insight into a possible function for miR-31 in natural Treg-mediated suppression. Future work should address whether miR-31 overexpression can reduce the suppressive activity of Tregs and their effect on the immune response against tumor cells [52]. In another example, recent data indicated that three members of the miR-148 family, including miR-148a, miR-148b, and miR-152, are important for dendritic cell (DC) maturation. DCs, which are another important component of the microenvironment, play an important role in linking innate and adaptive immune responsees and in tissue remodeling [53]. Subpopulations of DCs accumulate in solid tumors and induce immune tolerance, as well as suppress immune responses against tumors. MiR-148/152 regulate the innate response and antigen-presenting capacity of DCs and inhibit the production of cytokines, including IL-12, IL-6, TNF*α*, and IFN*β*. This effect is mediated by the direct targeting of calcium/calmodulin-dependent protein kinase II (CaMKII), which has been shown to be an important regulator of the maturation and function of DCs [53]. Thus, miRNAs may not only be a direct tumor suppressor, but may also inhibit tumorigenesis by enhancing the immune response.

## 4. MicroRNAs Affect the Extracellular Matrix Dynamics

The niche of a cancer cell plays important roles in the development of cancer. A major component of the niche is the extracellular matrix (ECM), which is commonly deregulated and becomes disorganized in diseases, especially cancer [54]. An abnormal ECM affects cancer progression through the deregulation of the behavior of stromal cells, facilitating tumor-associated angiogenesis and inflammation, and thus leading to the generation of a tumorigenic microenvironment. Consistent with the important roles of the ECM, multiple regulatory mechanisms exist to maintain normal ECM dynamics. The disruption of these control mechanisms deregulates and disorganizes the ECM, leading to cell invasion, migration, and angiogenesis. But what roles do miRNAs play in the ECM dynamics?

An important clue can be derived from ECM-degrading enzymes, which include MMPs. These proteases can have devastating destructive consequences on tissues and cause the demise of the whole organism. In cancer progression, the activities of these enzymes are important not only in the release of signaling molecules from the ECM, but also in promoting migration, invasion, and angiogenesis [54]. Interestingly, recent studies have demonstrated that a variety of miRNAs, acting as oncogenes or tumor suppressors, regulate survival and invasion by directly influencing the expression of MMPs [12] (Figure 1). For instance, lung metastasis from human osteosarcoma cells is correlated with the downregulation of miR-143, which promotes cellular invasion via MMP-13 upregulation [55]. In colorectal cancer, let-7c functions as a tumor metastasis suppressor by directly destabilizing the expression of MMP-11 [56]. Much like miR-143 and let-7c, low levels of miR-146b increase migration and invasion in glioma by directly upregulating MMP-16 expression [57]. In addition to the direct targeting of MMP promoters to participate in cancer progression, other miRNAs indirectly affect the expression levels of MMPs through different regulatory mechanisms. miR-520c and miR-373 upregulate MMP-9 expression and enhance human fibrosarcoma cell migration and growth not by direct binding to the MMP-9 promoter, but by directly targeting the 3′-UTR of the mRNAs of mTOR and SIRT1, which are negative regulators of MMP-9 expression [58].

Not surprisingly, tissue inhibitors of MMPs (TIMPs) also play an important role in carcinogenesis. The association of several miRNAs with TIMPs in relationship to cancer has recently been reported. A good example is miR-21, whose role as a major player in multiple high-migration and invasion cancers, including breast cancer, gliomas, and cholangiocarcinomas has been demonstrated [9, 17, 18, 46–48, 59, 60]. Mechanistically, several studies have shown that the expression level of miR-21 is inversely correlated with that of two MMP inhibitors, RECK and TIMP3, which are suppressors of malignancy [12, 17]. Notably, the mechanism underlying the activity of miR-21 as a direct inhibitor of RECK and TIMP3 was substantiated by directly targeting the 3′-UTR of the mRNAs of the two transcripts [17]. Taken together, these data provide strong evidence for the role of miR-21 in altering the ECM niche of tumors, thus allowing invasion and metastasis.

Collectively, the data provided by several studies support multiple roles for miRNAs in ECM composition and remodeling during the progression of tumors. These findings indicate that miRNAs could be beneficial to the progression of tumors at many levels, including inhibition of TIMPs, alteration of ECM component secretion, and activation of MMPs. The vast number of miRNAs now known to be deregulated in disease suggests that the altered expression levels of these molecules may be utilized by primary tumors to further their destructive migratory and invasive properties.

## 5. Concluding Remarks

The local microenvironment, or niche, of a cancer cell plays a critical role in cancer development and progression. The progression of tumors is associated with changes in the niche that are significant determinants of the migration and invasion capacity of tumor cells. There is extensive interplay and crosstalk between tumor cells and their surrounding microenvironment, in which the incipient neoplasias recruit and activate stromal cell types that ultimately sustain metastatic dissemination. Therefore, understanding how the deregulation of tumor niches influences cancer progression may be of value for the development of therapeutic strategies for the control of tumor spread. As described, miRNAs act as managers of heterotypic signaling in CAFs and in the ECM, and influence the responses to hypoxia, providing a novel potential therapeutic target. Advances in the field of RNA-based drug design have indicated the potential of miRNA-mediated diagnostics and therapies. In the near future, targeting miRNAs in niches may provide yet another effective avenue to combat the complicated illness that is cancer.

## References

[1] Hanahan D, Weinberg RA (2011). Hallmarks of cancer: the next generation. *Cell*.

[2] Hanahan D, Weinberg RA (2000). The hallmarks of cancer. *Cell*.

[3] Egeblad M, Nakasone ES, Werb Z (2010). Tumors as organs: complex tissues that interface with the entire organism. *Developmental Cell*.

[4] Friedl P, Alexander S (2011). Cancer invasion and the microenvironment: plasticity and reciprocity. *Cell*.

[5] Orimo A, Gupta PB, Sgroi DC (2005). Stromal fibroblasts present in invasive human breast carcinomas promote tumor growth and angiogenesis through elevated SDF-1/CXCL12 secretion. *Cell*.

[6] Polyak K (2007). Breast cancer: origins and evolution. *Journal of Clinical Investigation*.

[7] Croce CM (2009). Causes and consequences of microRNA dysregulation in cancer. *Nature Reviews Genetics*.

[8] Dornan D, Settleman J (2010). Cancer: miRNA addiction—depending on life’s little things. *Current Biology*.

[9] Harris AL (2002). Hypoxia—a key regulatory factor in tumour growth. *Nature Reviews Cancer*.

[10] Kulshreshtha R, Ferracin M, Wojcik SE (2007). A microRNA signature of hypoxia. *Molecular and Cellular Biology*.

[11] Kulshreshtha R, Davuluri RV, Calin GA, Ivan M (2008). A microRNA component of the hypoxic response. *Cell Death and Differentiation*.

[12] Wentz-Hunter KK, Potashkin JA (2011). The role of miRNAs as key regulators in the neoplastic microenvironment. *Molecular Biology International*.

[13] Hua Z, Lv Q, Ye W (2006). Mirna-directed regulation of VEGF and other angiogenic under hypoxia. *PLoS ONE*.

[14] Hebert C, Norris K, Scheper MA, Nikitakis N, Sauk JJ (2007). High mobility group A2 is a target for miRNA-98 in head and neck squamous cell carcinoma. *Molecular Cancer*.

[15] Donker RB, Mouillet JF, Nelson DM, Sadovsky Y (2007). The expression of Argonaute2 and related microRNA biogenesis proteins in normal and hypoxic trophoblasts. *Molecular Human Reproduction*.

[16] Hwang-Verslues WW, Chang PH, Wei PC (2011). MiR-495 is upregulated by E12/E47 in breast cancer stem cells, and promotes oncogenesis and hypoxia resistance via downregulation of E-cadherin and REDD1. *Oncogene*.

[17] Gabriely G, Wurdinger T, Kesari S (2008). MicroRNA 21 promotes glioma invasion by targeting matrix metalloproteinase regulators. *Molecular and Cellular Biology*.

[18] Chan JA, Krichevsky AM, Kosik KS (2005). MicroRNA-21 is an antiapoptotic factor in human glioblastoma cells. *Cancer Research*.

[19] Babar IA, Czochor J, Steinmetz A (2011). Inhibition of hypoxia-induced miR-155 radiosensitizes hypoxic lung cancer cells. *Cancer Biology and Therapy*.

[20] Crosby ME, Devlin CM, Glazer PM, Calin GA, Ivan M (2009). Emerging roles of microRNAs in the molecular responses to hypoxia. *Current Pharmaceutical Design*.

[21] Crosby ME, Kulshreshtha R, Ivan M, Glazer PM (2009). MicroRNA regulation of DNA repair gene expression in hypoxic stress. *Cancer Research*.

[22] Volinia S, Calin GA, Liu CG (2006). A microRNA expression signature of human solid tumors defines cancer gene targets. *Proceedings of the National Academy of Sciences of the United States of America*.

[23] Camps C, Buffa FM, Colella S (2008). Hsa-miR-210 is induced by hypoxia and is an independent prognostic factor in breast cancer. *Clinical Cancer Research*.

[24] Porkka KP, Pfeiffer MJ, Waltering KK, Vessella RL, Tammela TLJ, Visakorpi T (2007). MicroRNA expression profiling in prostate cancer. *Cancer Research*.

[25] Foekens JA, Sieuwerts AM, Smid M (2008). Four miRNAs associated with aggressiveness of lymph node-negative, estrogen receptor-positive human breast cancer. *Proceedings of the National Academy of Sciences of the United States of America*.

[26] Lawrie CH, Gal S, Dunlop HM (2008). Detection of elevated levels of tumour-associated microRNAs in serum of patients with diffuse large B-cell lymphoma. *British Journal of Haematology*.

[27] Huang Q, Gumireddy K, Schrier M (2008). The microRNAs miR-373 and miR-520c promote tumour invasion and metastasis. *Nature Cell Biology*.

[28] Voorhoeve PM, le Sage C, Schrier M (2006). A genetic screen implicates miRNA-372 and miRNA-373 as oncogenes in testicular germ cell tumors. *Cell*.

[29] Place RF, Li LC, Pookot D, Noonan EJ, Dahiya R (2008). MicroRNA-373 induces expression of genes with complementary promoter sequences. *Proceedings of the National Academy of Sciences of the United States of America*.

[30] Calin GA, Dumitru CD, Shimizu M (2002). Frequent deletions and down-regulation of micro-RNA genes miR15 and miR16 at 13q14 in chronic lymphocytic leukemia. *Proceedings of the National Academy of Sciences of the United States of America*.

[31] Migliazza A, Bosch F, Komatsu H (2001). Nucleotide sequence, transcription map, and mutation analysis of the 13q14 chromosomal region deleted in B-cell chronic lymphocytic leukemia. *Blood*.

[32] Lagos-Quintana M, Rauhut R, Lendeckel W, Tuschl T (2001). Identification of novel genes coding for small expressed RNAs. *Science*.

[33] Klein U, Lia M, Crespo M (2010). The DLEU2/miR-15a/16-1 cluster controls B cell proliferation and its deletion leads to chronic lymphocytic leukemia. *Cancer Cell*.

[34] Yamakuchi M, Yagi S, Ito T, Lowenstein CJ (2011). Microrna-22 regulates hypoxia signaling in colon cancer cells. *PLoS ONE*.

[35] Xiong Y, Fang JH, Yun JP (2010). Effects of microrna-29 on apoptosis, tumorigenicity, and prognosis of hepatocellular carcinoma. *Hepatology*.

[36] Yamakuchi M, Lotterman CD, Bao C (2010). P53-induced microRNA-107 inhibits HIF-1 and tumor angiogenesis. *Proceedings of the National Academy of Sciences of the United States of America*.

[37] Xu Q, Liu LZ, Qian X (2012). MiR-145 directly targets p70S6K1 in cancer cells to inhibit tumor growth and angiogenesis. *Nucleic Acids Research*.

[38] Cha ST, Chen PS, Johansson G (2010). MicroRNA-519c suppresses hypoxia-inducible factor-1*α* expression and tumor angiogenesis. *Cancer Research*.

[39] Kulshreshtha R, Ferracin M, Negrini M, Calin GA, Davuluri RV, Ivan M (2007). Regulation of microRNA expression: the hypoxic component. *Cell Cycle*.

[40] de Wever O, Mareel M (2003). Role of tissue stroma in cancer cell invasion. *Journal of Pathology*.

[41] Aboussekhra A (2011). Role of cancer-associated fibroblasts in breast cancer development and prognosis. *International Journal of Developmental Biology*.

[42] Kurose K, Gilley K, Matsumoto S, Watson PH, Zhou XP, Eng C (2002). Frequent somatic mutations in PTEN and TP53 are mutually exclusive in the stroma of breast carcinomas. *Nature Genetics*.

[43] Hill R, Song Y, Cardiff RD, Van Dyke T (2005). Selective evolution of stromal mesenchyme with p53 loss in response to epithelial tumorigenesis. *Cell*.

[44] Qiu W, Hu M, Sridhar A (2008). No evidence of clonal somatic genetic alterations in cancer-associated fibroblasts from human breast and ovarian carcinomas. *Nature Genetics*.

[45] Yamamichi N, Shimomura R, Inada KI (2009). Locked nucleic acid in situ hybridization analysis of miR-21 expression during colorectal cancer development. *Clinical Cancer Research*.

[46] Rask L, Balslev E, Jørgensen S (2011). High expression of miR-21 in tumor stroma correlates with increased cancer cell proliferation in human breast cancer. *APMIS*.

[47] Lee JA, Lee HY, Lee ES (2011). Prognostic implications of microRNA-21 overexpression in invasive ductal carcinomas of the breast. *Journal of Breast Cancer*.

[48] Giovannetti E, Funel N, Peters GJ (2010). MicroRNA-21 in pancreatic cancer: correlation with clinical outcome and pharmacologic aspects underlying its role in the modulation of gemcitabine activity. *Cancer Research*.

[49] Aprelikova O, Yu X, Palla J (2010). The role of miR-31 and its target gene SATB2 in cancer-associated fibroblasts. *Cell Cycle*.

[50] Musumeci M, Coppola V, Addario A (2011). Control of tumor and microenvironment cross-talk by miR-15a and miR-16 in prostate cancer. *Oncogene*.

[51] Png KJ, Halberg N, Yoshida M, Tavazoie SF (2012). A microRNA regulon that mediates endothelial recruitment and metastasis by cancer cells. *Nature*.

[52] Rouas R, Fayyad-Kazan H, El Zien N (2009). Human natural Treg microRNA signature: role of microRNA-31 and microRNA-21 in FOXP3 expression. *European Journal of Immunology*.

[53] Liu X, Zhan Z, Xu L (2010). MicroRNA-148/152 impair innate response and antigen presentation of TLR-triggered dendritic cells by targeting CaMKII*α*. *Journal of Immunology*.

[54] Lu P, Weaver VM, Werb Z (2012). The extracellular matrix: a dynamic niche in cancer progression. *The Journal of Cell Biology*.

[55] Osaki M, Takeshita F, Sugimoto Y (2011). MicroRNA-143 regulates human osteosarcoma metastasis by regulating matrix metalloprotease-13 expression. *Molecular Therapy*.

[56] Han HB, Gu J, Zuo HJ (2012). Let-7c functions as a metastasis suppressor by targeting MMP11 and PBX3 in colorectal cancer. *Journal of Pathology*.

[57] Xia H, Qi Y, Ng SS (2009). microRNA-146b inhibits glioma cell migration and invasion by targeting MMPs. *Brain Research*.

[58] Liu P, Wilson MJ (2012). miR-520c and miR-373 upregulate MMP9 expression by targeting mTOR and SIRT1, and activate the Ras/Raf/MEK/Erk signaling pathway and NF-*κ*B factor in human fibrosarcoma cells. *Journal of Cellular Physiology*.

[59] Yan LX, Huang XF, Shao Q (2008). MicroRNA miR-21 overexpression in human breast cancer is associated with advanced clinical stage, lymph node metastasis and patient poor prognosis. *RNA*.

[60] Selaru FM, Olaru AV, Kan T (2009). MicroRNA-21 is overexpressed in human cholangiocarcinoma and regulates programmed cell death 4 and tissue inhibitor of metalloproteinase 3. *Hepatology*.

